# The curse of the prey: *Sarcoptes *mite molecular analysis reveals potential prey-to-predator parasitic infestation in wild animals from Masai Mara, Kenya

**DOI:** 10.1186/1756-3305-4-193

**Published:** 2011-10-06

**Authors:** Francis Gakuya, Luca Rossi, Jackson Ombui, Ndichu Maingi, Gerald Muchemi, William Ogara, Ramón C Soriguer, Samer Alasaad

**Affiliations:** 1 Department of Veterinary and Capture Services, Kenya Wildlife Service, Kenya; 2 Dipartimento di Produzioni Animali, Epidemiologia ed Ecologia, Università degli Studi di Torino, Via Leonardo da Vinci 44, I-10095, Grugliasco, Italy; 3 Department of Public Health, Pharmacology & Toxicology, University of Nairobi, Kenya; 4 Department of Pathology and Microbiology, University of Nairobi, Kenya; 5 Estación Biológica de Doñana, Consejo Superior de Investigaciones Científicas (CSIC), Avda. Américo Vespucio s/n 41092 Sevilla, Spain; 6 Institute of Evolutionary Biology and Environmental Studies (IEU), University of Zürich, Winterthurerstrasse 190, 8057 Zürich, Switzerland

**Keywords:** *Sarcoptes scabiei*, microsatellites, genetic structure, gene flow, cheetah, lion, wildebeest, Thomson's gazelle, favourite prey

## Abstract

**Background:**

Recently, there have been attempts to understand the molecular epidemiology of *Sarcoptes scabiei*, to evaluate the gene flow between isolates of *S. scabiei *from different hosts and geographic regions. However, to our knowledge, a molecular study has not been carried out to assess the molecular diversity and gene flow of *Sarcoptes *mite in a predator/prey ecosystem.

**Results:**

Our study revealed an absence of gene flow between the two herbivore (Thomson's gazelle and wildebeest)- and between the two carnivore (lion and cheetah)-derived *Sarcoptes *populations from Masai Mara (Kenya), which is in discrepancy with the host-taxon law described for wild animals in Europe. Lion- and wildebeest-derived *Sarcoptes *mite populations were similar yet different from the Thomson's gazelle-derived *Sarcoptes *population. This could be attributed to *Sarcoptes *cross-infestation from wildebeest ("favourite prey") of the lion, but not from Thomson's gazelle. The cheetah-derived *Sarcoptes *population had different subpopulations: one is cheetah-private, one similar to the wildebeest- and lion-derived *Sarcoptes *populations, and another similar to the Thomson's gazelle-derived *Sarcoptes *mite population, where both wildebeest and Thomson's gazelle are "favourite preys" for the cheetah.

**Conclusions:**

In a predator/prey ecosystem, like Masai Mara in Kenya, it seems that *Sarcoptes *infestation in wild animals is prey-to-predator-wise, depending on the predator's "favourite prey". More studies on the lion and cheetah diet and behaviour could be of great help to clarify the addressed hypotheses. This study could have further ramification in the epidemiological studies and the monitoring protocols of the neglected *Sarcoptes *mite in predator/prey ecosystems.

## Background

*Sarcoptes scabiei *is a ubiquitous ectoparasite infecting more than 100 species of mammals, worldwide [1-3].

An epidemic can result from the introduction of a single case of scabies into crowded living conditions [4], which may result in devastating mortality in wild and domestic animals [5], with huge economic losses affecting the world animal trade [6].

Numerous epidemiological studies have been reported from different human, wild and domestic populations [7,8] but the epidemiology of sarcoptic mange is still not well understood and seems to differ between different areas and animal species of the world [1].

Recently, there have been attempts to understand the molecular epidemiology of the *Sarcoptes *mite, to evaluate the gene flow between isolates of *S. scabiei *from different hosts and geographic regions [9]. Walton et al. [10,11], used multi-locus genotyping applied to microsatellite markers to substantiate previous findings to the effect that gene flow between scabies mite populations in human and dog hosts is extremely rare in northern Australia. Genetic differences were detected between geographically distinct populations, and even between different people in the same household. Microsatellite markers were used by Alasaad et al. [12] to describe a new phenomenon of genetic structuring among *S. scabiei *at the individual host skin-scale level. Host-taxon law (carnivore-, omnivore- and herbivore-derived *Sarcoptes *mite populations) was established for *Sarcoptes *mite populations in wild animals from Europe [13,14]. However, to our knowledge, a molecular study has not been carried out to assess the molecular diversity and gene flow of *Sarcoptes *mite in a predator/prey ecosystem.

Sarcoptic mange has continuously threatened wildlife populations in most of the wildlife areas in Kenya. One of the animals that, to date, has remained a preferential host for *Sarcoptes *mite is the cheetah (*Acinonyx jubatus*). The cheetah population in Kenya is estimated to be less than 1000 individuals [15]. The cheetah is now extinct in some areas within its historical and geographical range, and the remaining population is highly endangered. Among the major factors thought to have brought about the decline of the cheetah are diseases, with sarcoptic mange being placed among the leading causes of death [16]. *Sarcoptes *mite also affects Thomson gazelles (*Gazelle thompsonii*), the most important prey animal of the cheetah, and wildebeest (*Connochaetes taurinus*), another prey species of lions (*Panthera leo*) and even cheetah [17].

## Results

Twenty six alleles were detected from the eight microsatellite loci. The allele count for each of the 8 loci ranged from one (Sarms36 and Sarms40, which were excluded from the analyses) to seven (Sarms34). Eleven private alleles (alleles present in only one host-derived population) were detected. The number of private alleles ranged between zero (Sarms35) and three (Sarms34 and Sarms44). The wildebeest-derived *Sarcoptes *population had the highest number, with five private alleles, followed by the cheetah with three and lion with two private alleles, while only one was detected from the Thomson's gazelle-derived mite population (Table 1).

**Table 1:** Private alleles detected at the eight microsatellite loci of the four wildlife-derived *Sarcoptes *mite populations from Masai Mara in Kenya, together with their overall frequencies

Locus	Allele	Overall frequency	Animal-derived *Sarcoptes *population
	174	0.0088	Thomson's gazelle
	
Sarms34	204	0.0175	Wildebeest
	
	206	0.1404	Cheetah

Sarms37	176	0.0093	Wildebeest
	
	180	0.0463	Cheetah

Sarms41	236	0.0085	Cheetah
	
	240	0.0085	Lion

	270	0.0086	Lion
	
Sarms44	276	0.0172	Wildebeest
	
	279	0.0172	Wildebeest

Sarms45	172	0.0086	Wildebeest

For all loci examined there was no evidence of linkage disequilibrium (*P *> 0.05). Deviation from Hardy-Weinberg equilibrium (HWE) was detected in Sarms34 (*p *= 0.008), Sarms35 (*p *= 0.014) and Sarms36 (*p *< 0.001) in Thomson's gazelle-derived *Sarcoptes *population, and in Sarms44 (*p *= 0.02) in cheetah-derived *Sarcoptes *mite population.

Intra-host variation was detected in all the animals from Thomson's gazelle-, wildebeest-, and cheetah-derived *Sarcoptes *populations, and only in one animal in lion-derived *Sarcoptes *mite population.

Mean number of alleles was 1.625 ± 0.992 (for Thomson's gazelle-), 2.375 ± 1.409 (for wildebeest-), 2 ± 0.866 (for cheetah-), and 1.875 ± 0.927 (for lion-derived *Sarcoptes *mite population). Mean expected heterozygosity was 0.20115 ± 0.26909 (for Thomson's gazelle-), 0.3228 ± 0.31033 (for wildebeest-), 0.2735 ± 0.23382 (for cheetah-), and 0.26655 ± 0.27041 (for lion-derived *Sarcoptes *mite population).

AMOVA analysis showed differentiation among populations (*F_ST _*= 0.24241; *P *< 0.001), which indicates that the mite component populations differed greatly. *F_ST _*value between all wild animal-derived *Sarcoptes *mite populations was statistically supported, with the exception of lion- and wildebeest-derived *Sarcoptes *mite populations, where *F_ST _*value was not statistically supported. The highest *F_ST _*value was between Thomson's gazelle- and lion-derived *Sarcoptes *mite populations (Table 2).

**Table 2:** Matrix of fixation index (*F_ST_*) significant *P *values, with significance level P = 0.05 (above diagonal), and population pairwise *F_ST _*(below diagonal) for each pairwise comparison of four *Sarcoptes *mite populations from Masai Mara, Kenya

	Thomson's gazelle	Cheetah	Wildebeest	Lion
**Thomson's gazelle**	_	< 0.001*	< 0.001*	< 0.001*

**Cheetah**	0.23386	_	< 0.001*	< 0.001*

**Wildebeest**	0.33334	0.19637	_	0.18018

**Lion**	0.41466	0.15064	0.02921	_

These results were confirmed by the average number of pairwise differences (PXY) between *Sarcoptes *populations, which was statistically supported between all pairs of *Sarcoptes *populations with the exception of wildebeest- and lion-derived *Sarcoptes *mite populations (Table 3).

**Table 3:** Population average pairwise differences between four *Sarcoptes *mite derived populations from Masai Mara, Kenya

	Thomson's gazelle	Cheetah	Wildebeest	Lion
**Thomson's gazelle**	1.16538	1.75850	2.31607	2.08393

**Cheetah**	0.41662	1.51837	2.20000	1.68000

**Wildebeest**	0.67843	0.38587	2.10989	1.76020

**Lion**	0.84739	0.26697	0.05141	1.30769

Above diagonal: average number of pairwise differences between populations (PiXY). Diagonal elements: average number of pairwise differences within population (PiX). Below diagonal: corrected average pairwise difference (PiXY-(PiX+PiY)/2).

The number of migrations (M = 2 Nm for diploid data) ranged from 0.70582 (between Thomson's gazelle- and lion-derived *Sarcoptes *mite populations) and 16.61832 (between wildebeest- and lion-derived *Sarcoptes *populations). The effective number of migrations per generation was lower between the two herbivore (Thomson's gazelle and wildebeest)-derived *Sarcoptes *populations (0.99996), compared with the two carnivore (lion and cheetah)-derived mite populations (2.8191). The cheetah-derived *S. scabiei *population showed a relatively high migration rate with all the studied populations (for more details see Table 4).

**Table 4:** Matrix of number of effective migrants per generation (Nm) for each pairwise comparison of the four *Sarcoptes *mite populations from Masai Mara (Kenya)

	Thomson's gazelle	Cheetah	Wildebeest	Lion
**Thomson's gazelle**				

**Cheetah**	1.63799			

**Wildebeest**	0.99996	2.04621		

**Lion**	0.70582	2.81910	16.61832	

**Figure 1:**
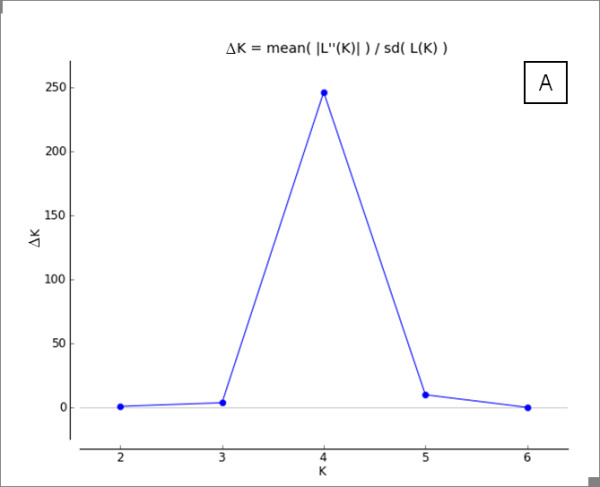
**Results of STRUCTURE analysis of the four studied *Sarcoptes *mite populations**. Results of STRUCTURE analysis of the four studied *Sarcoptes *mite populations from Masai Mara (Kenya) showing ΔK as proposed by Evanno et al. [18] method (1-10 clusters modelled). The best fit of the data was four clusters.

**Figure 2:**
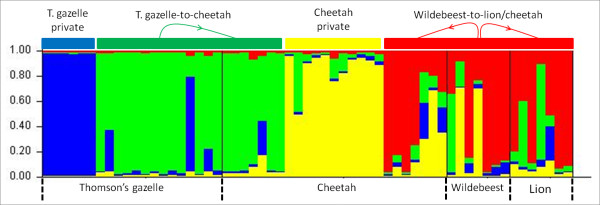
**Bar plot of the degree of individual variation between fifty nine *S. Scabiei***. Bar plot of the degree of individual variation between fifty nine *S. scabiei *from different host species in the Masai Mara (Kenya) assigned to given genetic clusters in STRUCTURE, when five (K = 4) populations are assumed in the dataset. Each cluster is represented by a different colour.

Using the Bayesian assignment test of the software STRUCTURE, ln Pr(X|K) for the likely number of populations K, it was consistently found K = 4, and we obtained the same results when applying Evanno et al. [18] criteria (Figure 1). Considering K = 4, herbivore (Thomson's gazelle and wildebeest)-derived *Sarcoptes *mite populations were separated into two different clusters, while carnivore (lion and cheetah)-derived *Sarcoptes *mite populations were more similar. The lion-derived *Sarcoptes *mite population clustered with the wildebeest-derived *Sarcoptes *mite population. Cheetah-derived *Sarcoptes *mite population had three different subpopulations: one subpopulation was similar to Thomson's gazelle-derived *Sarcoptes *mite population, one was similar to wildebeest- and lion-derived *Sarcoptes *mite populations, and one was cheetah-private. The Thomson's gazelle-derived *Sarcoptes *population had two subpopulations: one similar to cheetah-derived *Sarcoptes *population and another private one (Figure 2).

**Figure 3:**
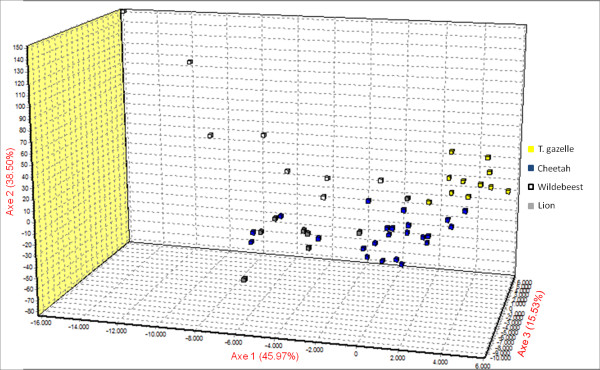
**Factorial Component Analysis (FCA)**. Factorial Component Analysis (FCA) of the proportion of variation of four *Sarcoptes *mite populations from Masai Mara (Kenya) assigned to given genetic clusters in Genetix.

The scatter plot of the Factorial Component Analysis (FCA), for the individuals of *S. scabiei *collected from the sympatric wild animals in Kenya, confirmed the results obtained by the Bayesian assignment test: Thomson's gazelle- and wildebeest-derived *Sarcoptes *individuals were scattered separately. Wildebeest- and lion-derived *Sarcoptes *individuals were similar to each other. Cheetah-derived *Sarcoptes *individuals were the most diverse ones, distributed between Thomson's gazelle-, lion- and wildebeest-derived *Sarcoptes *individuals (Figure 3).

## Discussion

As with other highly divergent taxa, with *Sarcoptes scabiei *few loci and low sample sizes are deemed sufficient to find strong population differentiation between host species [11,14]. The unusually high number of private alleles detected in all populations studied was the first indicator of the genetic separation and current lack of gene flow between *Sarcoptes *mite populations in Masai Mara.

The mean number of alleles and the mean expected heterozygosity were relatively high in wildebeest- and cheetah-derived *Sarcoptes *mite populations, compared to lion- and Thomson's gazelle-derived *Sarcoptes *mite populations. This could be attributed (i) to putative higher resistance to parasite infestation [13,19], (ii) to a wider range of geographical movement especially in the case of wildebeest [20-23], (iii) and/or to a higher diversity of prey species in the case of cheetah compared with lion [24-26]. Lions and Thomson's gazelles have smaller home ranges, which is limited by food availability [27,28]. These results were confirmed by the detection of infra-host variations and the deviation from HWE in some loci from the corresponding mite populations, which could be attributed to the presence of subpopulations [12,29]. *Sarcoptes *mites lack free-living stages, and individual hosts, depending on their susceptibility and behaviour, are essentially ephemeral habitats providing patchy environments that hamper random mating [13,30,31].

AMOVA analysis (showing differentiation among populations) confirmed the presence of genetic differentiation and the absence of gene flow between wildlife-derived *Sarcoptes *mite populations from Masai Mara, with the exception of lion- and wildebeest-derived *Sarcoptes *mite populations. This was confirmed by the Bayesian assignment test which separated the two herbivore (Thomson's gazelle and wildebeest)- and two carnivore (lion and cheetah)-derived *Sarcoptes *populations into different clusters, which is not in concordance with the host-taxon phenomenon described for wild animals in Europe [13,14].

All AMOVA analysis, the Bayesian assignment test (between mites), the scatter plot of the FCA (for the individuals *Sarcoptes *mite), and the effective number of migrations per generation showed that lion- and wildebeest-derived *Sarcoptes *mite populations were both similar but different from the Thomson's gazelle-derived *Sarcoptes *population. This could be attributed to *Sarcoptes *cross-infestation from wildebeest to lion, but not from Thomson's gazelle to lion. The cheetah-derived *Sarcoptes *population has different subpopulations: one which is private, one similar to wildebeest- and lion-derived *Sarcoptes *populations, and another similar to Thomson's gazelle-derived *Sarcoptes *mite population, which could be attributed to the diet preference, since the cheetah is known to prey upon Thomson's gazelle and wildebeest, especially calves [24], in contrast to lions which are known to prey on wildebeest and rarely Thomson's gazelle [25,26]. There is a high probability of *Sarcoptes *mite transmission from the prey to the predator during the hunting process and during feeding which can lead to prey-to-predator *Sarcoptes *gene flow via direct transfer of *S. scabiei*, depending on the predator's "favourite prey". This phenomenon explains the existence of gene flow of *Sarcoptes *mite between lion and wildebeest, the cheetah and Thomson gazelle, and the cheetah and wildebeest. Lions preying on Thomson gazelles is quite rare especially in areas where there are other big game species [25], like in the Masai Mara ecosystem [32].

In the study reported by Rasero et al. [13] on *Sarcoptes *mite genetic diversity from wild animals in Europe, there was lack of interaction between carnivore, herbivore and omnivore hosts, while in a predator/prey ecosystem like Masai Mara there is evidence of such an interaction, which could lead to alterations in host-taxon phenomenon on prey-to-predator gene flow. Moreover, lions and cheetahs may preferentially select mangy preys, since the affected preys could have a reduced flight response compared with healthy individuals.

## Conclusions

Bearing in mind some limitations of our study regarding the sampling size of the studied wild animals and the microsatellite panel used, our study revealed alteration in the specificity of *Sarcoptes *mite by its host-taxon. This alteration could be at least partially explained by predator/prey interactions. In a predator/prey ecosystem, like Masai Mara in Kenya, it seems that *Sarcoptes *infestation is from prey-to-predator, in relation to the predator's "favourite prey". More studies on the lion and the cheetah diet and their behaviour could be of great help to clarify the hypotheses addressed in our study, as well as more studies on *Sarcoptes *mites from: i) the same carnivore hosts and their "favourite preys" in different ecosystems; and ii) additional carnivore hosts (both predators and scavengers) with broader and narrower prey spectra. This study could have further ramification in the epidemiological studies and the monitoring protocols of the neglected *Sarcoptes *mite in predator/prey ecosystems. The effective control of sarcoptic mange in the threatened carnivores, as it the case of the cheetah in Masai Mara (which is estimated at only 61 animals; [33]), should take into account the management and control of *S. scabiei *in its favourite preys.

## Methods

### Masai Mara ecosystem

This study was carried out in the Masai Mara National Reserve and Mara Conservancy from the protected area of the Masai Mara ecosystem (1013' and 1045' S, and 34045' and 35025 E), which covers approximately 1,850 km^2 ^and is located at the northern tip of the Serengeti National Park (Tanzania). Rainfall increases along a southeast-northwest gradient. The terrain of the reserve is primarily open grassland with seasonal riverlets.

### Ethical approval

The Committee of the Department of Veterinary and Capture Services, Kenya Wildlife Service (KWS) approved this study including the animal protocols. KWS guidelines on Wildlife Veterinary Practice-2006 were used. All veterinaries in KWS were guided by Veterinary Surgeons Act Cap 366 Laws of Kenya that regulates veterinary practice in Kenya.

### Specimen collection and DNA extraction

Between 2007 and 2009 mangy animals, based on clinical observation (pruritus, alopecia, crust formation, skin roughening and poor body condition) were chemically immobilized, through darting using etorphine hydrochloride (M99^® ^9.8 mg/ml, Novartis South Africa Pty Ltd, Isando, South Africa) combined with Xylazine hydrochloride (Ilium Xylazil-100 100 mg/ml, Troy Laboratories Pty Ltd, Smithfield, Australia). The most affected area of skin was scraped with a scalpel until it bled in order to obtain hairs and crusts for parasitological examination. The scrapings were placed in universal bottles containing 70% ethanol and transported to the laboratory [34]. A total of fifty nine adult mites were collected: twenty *Sarcoptes *mites from three Thomson's gazelles (*Eudorcas thompsonii*), seven from three wildebeests (*Connochaetes taurinus*), twenty five from three cheetahs (*Acinonyx jubatus*), and seven from three lions (*Panthera leo*).

All mites were identified as *S. scabiei *on the basis of known morphological criteria [35]. The DNA of individual *Sarcoptes *mites was extracted using the HotSHOT Plus ThermalSHOCK technique [36]. Two blanks (reagents only) were included in each extraction to monitor for contamination.

### Fluorescent-based Polymerase chain reaction (PCR) analysis of microsatellite DNA

As described by Alasaad et al. [12], eight specific *Sarcoptes *mite microsatellites (Sarms 34-37, 40, 41, 44 and 45) were used with one 8× multiplex PCR. One primer from each set was 5' labelled with 6-FAM, VIC, NED or PET^® ^fluorescent dye tag (Applied Biosystems, Foster City, CA, USA). Each 15 μl PCR reaction mixture consisted of 3 μl of the single mite DNA, together with the PCR mixture containing all primer pairs (ranging from 0.04 to 0.1 μM per primer), 200 μM of each dNTP, 1.5 μl of 10× PCR buffer (200 mM KCl and 100 mM Tris-HCl, pH 8.0), 1.5 mM MgCl2 and 0.15 μl (0.5 U/reaction) HotStar Taq (QIAGEN, Milano, Italy). The thermal profile in a 2720 thermal cycler (Applied Biosystems, Foster City, CA, USA) was as follows: 15 min at 95°C (initial denaturing), followed by 37 cycles of three steps of 30 s at 94°C (denaturation), 45 s at 55°C (annealing) and 1.5 min at 72°C (extension), before a final elongation of 7 min at 72°C. Fluorescent PCR amplification products were analysed using formamide with Size Standard 500 Liz (Applied Biosystems, Foster City, CA, USA) by ABI PRISM 310 Genetic Analyser with pop4. Allele calling was performed using the GeneMapper v. 4.0 software (Applied Biosystems, Foster City, CA, USA).

### Molecular analyses

Expected (*H_E_*) and observed (*H_O_*) heterozygosity, linkage disequilibria (LD), and HWE tests were calculated using GENEPOP (v.3.4; [37]). Deviations from HWE and tests for LD were evaluated using Fisher's exact tests and sequential Bonferroni corrections.

The heterogeneity of genetic diversity among the different *Sarcoptes *mite populations was estimated by the partition of variance components (AMOVA) applying conventional *F_ST _*statistics using allele's frequencies as implemented in Arlequin 3.11 [38]. The analysis of relationships between mites was carried out by the Bayesian assignment test of the software STRUCTURE (v.2.3.3; [39]). Burn-in and run lengths of Markov chains were both 100000. We ran 20 independent runs for each K (for K = 1-10). The most likely number of clusters was determined using two approaches; by estimating the posterior probability for each K as recommended by Pritchard et al. [39], and by using the method of Evanno et al. [18]. Finally, each of the inferred clusters was associated with the component populations of its mites.

The degree of genetic relationship among populations was further investigated with FCA as implemented in Genetix v.4.05.2 [40].

## Competing interests

The authors declare that they have no competing interests.

## Authors' contributions

FG, LR, JNO, NM, GM, WO, RS, and SA conceived and designed the experiments. FG and MO performed the field work experiments. FG and SA performed the molecular work. Manuscript was written by all co-authors. All authors read and approved the final manuscript.
